# Predicting Drug Resistance in *Mycobacterium tuberculosis*: A Machine Learning Approach to Genomic Mutation Analysis

**DOI:** 10.3390/diagnostics15030279

**Published:** 2025-01-24

**Authors:** Guillermo Paredes-Gutierrez, Ricardo Perea-Jacobo, Héctor-Gabriel Acosta-Mesa, Efren Mezura-Montes, José Luis Morales Reyes, Roberto Zenteno-Cuevas, Miguel-Ángel Guerrero-Chevannier, Raquel Muñiz-Salazar, Dora-Luz Flores

**Affiliations:** 1 Facultad de Ingeniería Arquitectura y Diseño, Universidad Autónoma de Baja California, Campus Ensenada, Ensenada 22860, Mexico; paredesg@uabc.edu.mx (G.P.-G.); perear@uabc.edu.mx (R.P.-J.); miguel.angel.guerrero.chevannier@uabc.edu.mx (M.-Á.G.-C.); 2 Escuela de Ciencias de la Salud, Universidad Autónoma de Baja California, Campus Ensenada, Ensenada 22890, Mexico; 3 Instituto de Investigaciones en Inteligencia Artificial, Universidad Veracruzana, Xalapa 91097, Mexico; heacosta@uv.mx (H.-G.A.-M.); emezura@uv.mx (E.M.-M.); 4 Centro de Investigación en Alimentos y Desarrollo, Universidad Veracruzana, Xalapa 91190, Mexico; jluismorey@hotmail.com; 5 Instituto de Salud Pública, Universidad Veracruzana, Xalapa 91097, Mexico; rzenteno@uv.mx; 6 Red Multidisciplinaria de Investigación en Tuberculosis, Mexico City 22890, Mexico

**Keywords:** drug resistance, variant call format, extreme gradient boosting, *Mycobacterium tuberculosis*

## Abstract

**Background/Objectives:** Tuberculosis (TB), caused by *Mycobacterium tuberculosis* (*M. tuberculosis*), remains a leading cause of death from infectious diseases globally. The treatment of active TB relies on first- and second-line drugs, however, the emergence of drug resistance poses a significant challenge to global TB control efforts. Recent advances in whole-genome sequencing combined with machine learning have shown promise in predicting drug resistance. This study aimed to evaluate the performance of four machine learning models in classifying resistance to ethambutol, isoniazid, and rifampicin in *M. tuberculosis* isolates. **Methods:** Four machine learning models—Extreme Gradient Boosting Classifier (XGBC), Logistic Gradient Boosting Classifier (LGBC), Gradient Boosting Classifier (GBC), and an Artificial Neural Network (ANN)—were trained using a Variant Call Format (VCF) dataset preprocessed by the CRyPTIC consortium. Three datasets were used: the original dataset, a principal component analysis (PCA)-reduced dataset, and a dataset prioritizing significant mutations identified by the XGBC model. The models were trained and tested across these datasets, and their performance was compared using sensitivity, specificity, Precision, F1-scores and Accuracy. **Results:** All models were applied to the PCA-reduced dataset, while the XGBC model was also evaluated using the mutation-prioritized dataset. The XGBC model trained on the original dataset outperformed the others, achieving sensitivity values of 0.97, 0.90, and 0.94; specificity values of 0.97, 0.99, and 0.96; and F1-scores of 0.93, 0.94, and 0.92 for ethambutol, isoniazid, and rifampicin, respectively. These results demonstrate the superior accuracy of the XGBC model in classifying drug resistance. **Conclusions:** The study highlights the effectiveness of using a binary representation of mutations to train the XGBC model for predicting resistance and susceptibility to key TB drugs. The XGBC model trained on the original dataset demonstrated the highest performance among the evaluated models, suggesting its potential for clinical application in combating drug-resistant tuberculosis. Further research is needed to validate and expand these findings for broader implementation in TB diagnostics.

## 1. Introduction

TB is an infectious disease caused by bacteria from the *M. tuberculosis* complex. While primarily affecting the lungs, TB can manifest in other sites, such as the nervous system, bones, skin, intestines, genitals, and lymph nodes. Airborne transmission, like COVID-19, is the primary mode of spread, occurring through droplets expelled by TB patients. Approximately one-third of the global population is infected with *M. tuberculosis*. In 2023, TB led to 10.6 million illnesses and 1.6 million deaths, equivalent to 4500 daily fatalities [1]. The World Health Organization (WHO) estimated that globally, 558,000 cases (ranging from 483,000 to 639,000) of rifampicin-resistant TB (RR-TB), a potent first-line drug, occurred in 2021. Of these, 82% were multidrug-resistant TB (MDR-TB), indicating resistance to at least the second most crucial drug, isoniazid (H). Drug-resistant TB (DR-TB) cases have surged over the past 15 years, resulting in strains of *M. tuberculosis* resistant to all existing drugs, termed extensively drug-resistant TB (XDR-TB). DR-TB is transmitted similarly to drug-sensitive TB but is more complex to treat and expensive, and improperly managed cases can be potentially fatal [1].

Conventional DR-TB diagnosis relies on drug susceptibility testing (DST) through microbiological cultures, which takes 4 to 6 weeks to yield results. During this time, patients continue to transmit the bacteria due to a lack of appropriate drug treatment. Globally, only 64% of TB cases are diagnosed, leaving 3.6 million people untreated out of 10 million new cases, thereby contributing to further transmission [2]. Many countries, including Mexico, use acid-fast bacilli microscopy for TB diagnosis. This technique has been used for over a century but does not detect DR-TB.

Modern molecular methods offer significant advantages in scaling up DR-TB management and surveillance, providing rapid, standardized diagnostic assays with the potential for high performance and reduced laboratory biosafety requirements [3]. Molecular assays offer results within hours, with high sensitivity and specificity. The Xpert MTB/RIF assay is widely used for rapid *M. tuberculosis* identification and rifampicin (R) resistance detection, requiring just two hours; nevertheless, it only assesses R resistance. The new Xpert® MTB/XDR version identifies mutations linked to H, fluoroquinolones, second-line injectable drugs (amikacin, kanamycin, capreomycin), and ethionamide resistance in a single test [4].

Furthermore, recent advances in Next-Generation Sequencing (NGS) for *M. tuberculosis* have expedited the assay to 2–5 days, significantly lowering costs. This allows physicians to obtain a more comprehensive drug resistance (DR) profile within hours rather than weeks or months. Timely access to such information would facilitate tailored drug treatment and, consequently, earlier interruption of disease transmission [5]. Considering the above, an alternative to targeted mutation detection methods is NGS, which can identify common and rare mutations associated with anti-tuberculosis drug resistance [6]. Genes linked to drug resistance have been extensively studied, demonstrating that single-nucleotide polymorphisms (SNPs), deletions, and insertions (INDELs) lead to drug-resistant strains [7].

One promising path to tackle DR-TB is take advantage of machine learning (ML) and Deep Learning (DL) models for *M. tuberculosis* isolate classification and the prediction of drug resistance [8,9,10,11,12]. These models utilize algorithms capable of analyzing vast datasets and discerning intricate patterns, enabling more accurate identification of drug-resistant isolates from data sources like NGS.

This research aims to harness NGS-derived data of *M. tuberculosis* strains to train ML models for DR classification, surpassing the performance of whole-genome association studies (WGASs), in order to predict R, H, and ethambutol (E) drug resistance information.

## 2. Materials and Methods

### 2.1. Training Matrix

#### 2.1.1. Database

The dataset used for this study is derived from the Comprehensive Resistance Prediction for Tuberculosis: An International Consortium (CRyPTIC) and comprises genomic sequences from 12,289 *M. tuberculosis* clinical isolates collected globally [13]. These isolates, obtained from 23 countries, were selected to represent a broad spectrum of drug-resistant phenotypes, ensuring coverage across different lineages of the bacterium. The isolates underwent whole-genome sequencing and were tested for susceptibility to 13 anti-tubercular drugs, including first-line drugs such as R and H, second-line drugs like levofloxacin (Lfx) and amikacin (Am), and new or repurposed drugs such as bedaquiline (Bdq) and delamanid (Dlm).

The minimum inhibitory concentration (MIC) for each isolate was measured using a standardized microscale assay, ensuring uniform data collection across different laboratories. These phenotypic data were matched with whole-genome sequencing results, generating a VCF file for each isolate, facilitating in-depth genomic analysis. Notably, the dataset is enriched for rare resistance-associated variants, especially those linked to newly introduced drugs, allowing the exploration of resistance mechanisms beyond conventional drugs. In total, the dataset contains 6814 isolates resistant to at least one drug, including 2129 samples classified as R-resistant, multidrug-resistant, pre-extensively drug-resistant, or extensively drug-resistant.

#### 2.1.2. Data Preprocressing

The original VCF file contained mutation data for each isolate; however, its structure was not optimal for efficiently creating a training matrix. To address this issue and ensure computational feasibility, a preprocessing step was performed, which involved extracting a subset of 929 unique IDs from the original dataset of 12,289 isolates. This subset was selected as the maximum number of samples that could be processed within the available computational resources. While the selection was not based on specific sampling criteria, it aimed to maintain a balance that reasonably represented the diversity and variability of the original dataset.

**Figure 1:**
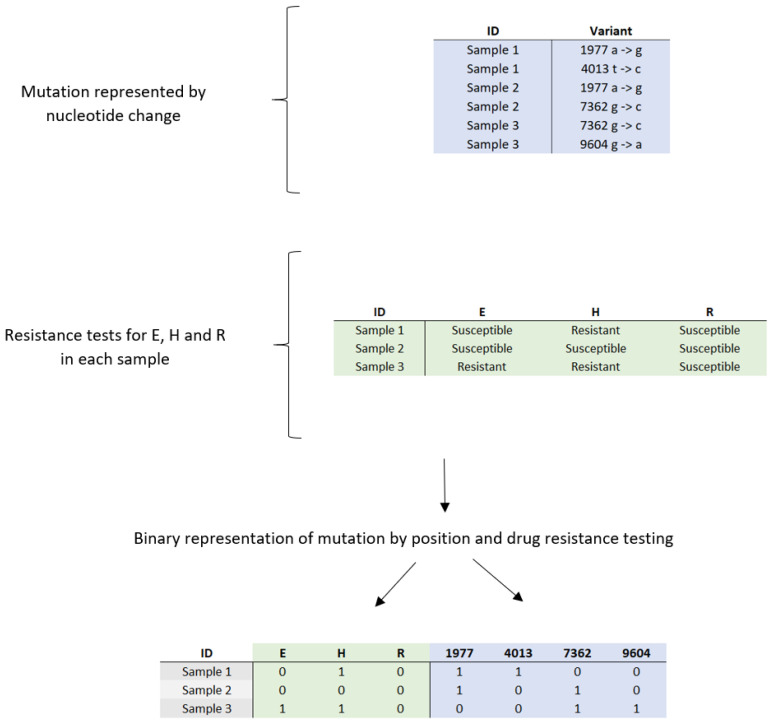
Conversion of categorical data on genetic mutations and drug sensitivity and resistance profiles in clinical samples to binary values: E = ethambutol; H = isoniazid; R = rifampicin.

During preprocessing, mutation information was reorganized to represent each mutation as a binary presence/absence indicator rather than a nucleotide change (Figure 1). Following this extraction, additional filtering was performed to exclude isolates lacking complete drug susceptibility data for E, H, and R. This refinement resulted in a final dataset of 847 IDs, representing a reduction of 8.8% from the initially extracted subset.

The final dataset comprised 79,256 unique mutations, which were carefully filtered to ensure accuracy and reliability for model training. Prioritizing data quality over quantity was essential, as models trained on incomplete or inaccurate data could produce unreliable predictions in clinical applications. Ultimately, the resulting training matrix consisted of 847 isolates, of which 589 were susceptible and 258 were resistant. A breakdown of the resistance data revealed 155 isolates resistant to E, 244 to H, and 200 to R.

**Figure 2:**
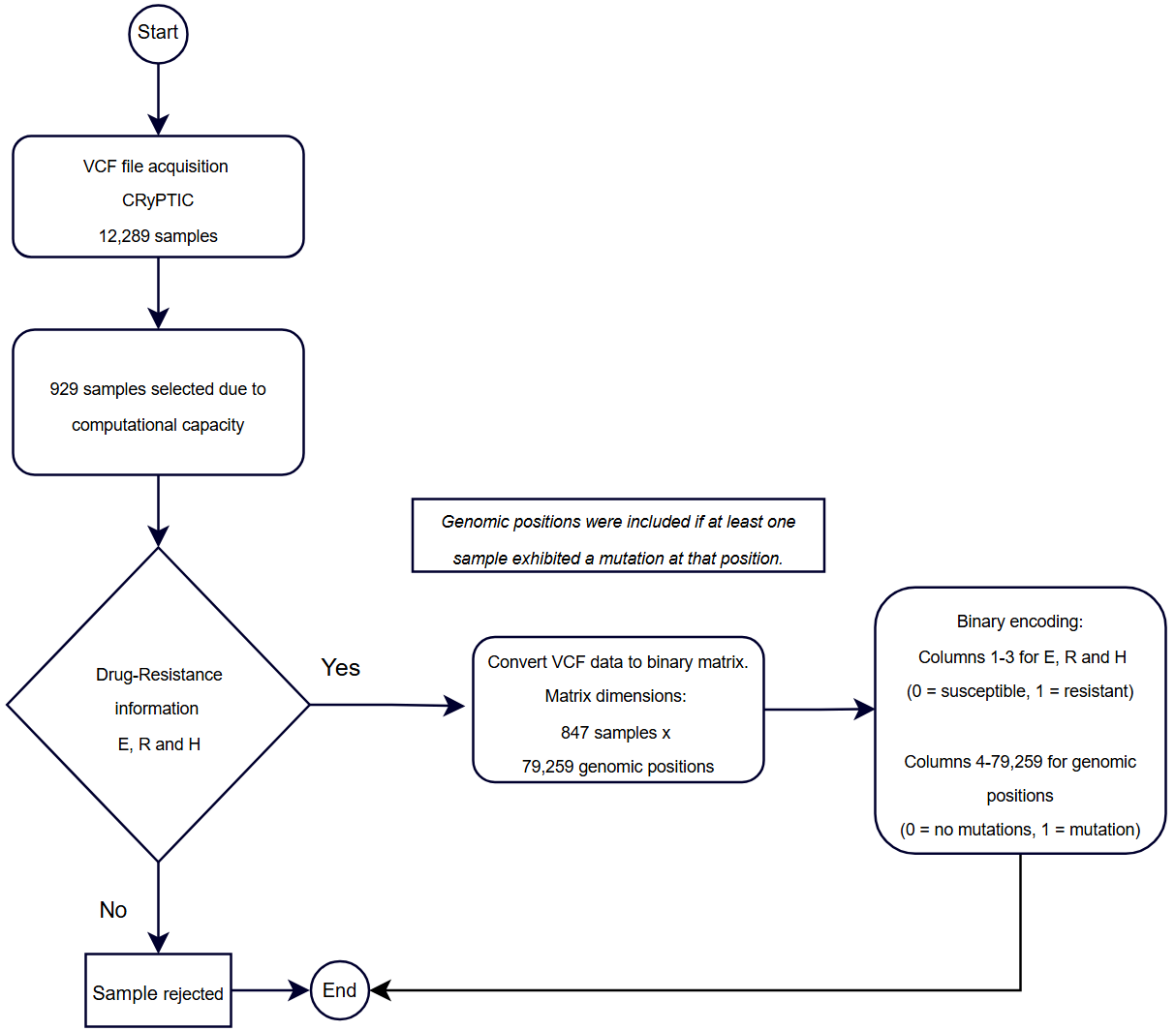
Workflow for sample selection, data processing, and binary representation for training matrix construction.

The training matrix was structured as (847 × 79,259), where the first three columns represented the drug resistance labels, serving as the target variables, while the remaining columns contained the unique mutations identified as predictors. This carefully curated dataset was used for training the machine learning models in this study, ensuring both computational manageability and high-quality data for predictive modeling tasks (Figure 2).

#### 2.1.3. Data Splitting: Training and Testing

To evaluate model performance and ensure its ability to generalize to unseen data, cross-validation was implemented. This technique is crucial for preventing issues such as overfitting, as it allows the model to be tested on different subsets of the original dataset.

Specifically, the cross_val_score() function from the Scikit-learn library (version 1.6.0) was used to perform cross-validation, implemented in Python 3.10.12. The dataset was initially divided into 677 training samples and 170 test samples. Cross-validation was applied exclusively to the 677 training samples. The process involved splitting this training set into 10 subsets or “folds” (k = 10). In each iteration, one fold (approximately 68 samples) was used as the test set while the remaining nine folds (approximately 609 samples) were used for training. This procedure was repeated 10 times, ensuring that each subset of the data was used as the test set once.

After all iterations, performance metrics, such as accuracy or loss, were averaged across the 10 iterations, providing a more robust and reliable evaluation of the model. Beyond performance evaluation, cross-validation also optimized the model’s hyperparameters, ensuring the best possible performance without sacrificing generalization ability.

#### 2.1.4. Dimensionality Reduction

**Figure 3:**
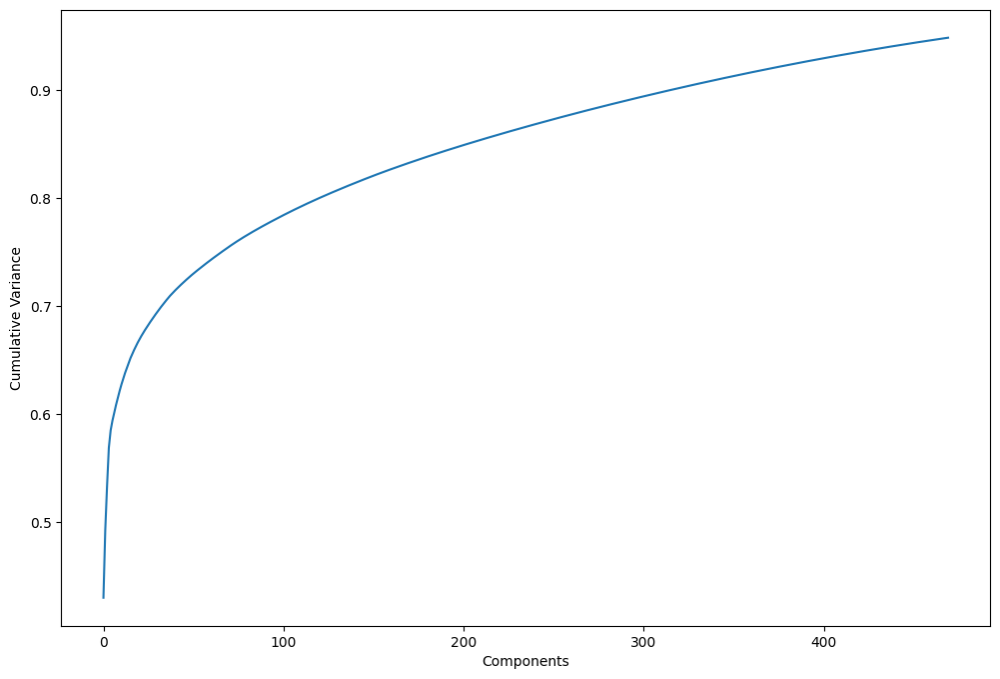
Increase in cumulative variance by using more principal components. In total, 470 were used to represent variance of 0.95.

Principal component analysis (PCA) was employed as an optimal transformation method for dimensionality reduction, minimizing squared errors. PCA focuses on identifying the features with the greatest variance and retaining the lower-order principal components that are most relevant to the dataset. In this study, 470 principal components were retained, representing 95% of the cumulative variance in the dataset (Figure 3). This approach allowed for a more compact representation of the data while preserving the most important features for analysis. The dataset reduced by PCA was subsequently used for all models evaluated in this study.

#### 2.1.5. Arbitrary Variant Reduction

The XGBC and LGBC models utilize the importance_type parameter, defined as ‘weight’, which measures how often a feature (in this case, a mutation) is used to split the data across decision trees. This analysis allowed for the identification of the most critical mutations, which were then compared against known resistance mutation catalogs. The feature reduction was performed based on these key mutations, generating a new dataset that prioritized the most important mutations identified by the best-performing model, XGBC, which outperformed the LGBC in predictive capability. This new dataset, referred to as RA (where ‘RA’ stands for ‘reducción arbitraria’ in Spanish), highlighting the arbitrary reduction in variants, was exclusively utilized with the XGBC model.

### 2.2. Machine Learning Models

This study employs a range of models, including the XGBoost Classifier (XGBC), LightGBM Classifier (LGBC), Gradient Boosting Classifier (GBC), and Multi-layer Perceptron Classifier (MLPClassifier, referred to as an artificial neural network or ANN). The XGBC model was applied to the original dataset as well as to PCA-reduced and RA datasets. In contrast, the other models were tested exclusively on the original and PCA-reduced datasets. These selections were made based on the models’ capabilities to process complex datasets and their effectiveness in addressing non-linear relationships.

XGBC and LGBC are gradient-boosting algorithms that exhibit exceptional performance in classification tasks, particularly when utilized with large datasets. These algorithms effectively address overfitting and enhance predictive accuracy through the application of advanced boosting techniques. In this context, a binary representation for mutations was employed, which does not account for the specific nucleotide changes associated with each mutation. Therefore, these models are particularly well suited for this analysis due to their decision tree-based framework. By leveraging the strengths of decision trees, XGBC and LGBC are capable of identifying significant mutations as they consistently appear across multiple decision paths within the ensemble. This capability provides valuable insights into the most critical genomic positions [14].

The inclusion of GBC is warranted due to its robust predictive performance and flexibility in optimizing various loss functions. The MLPClassifier, a neural network-based model, was chosen for its ability to capture intricate patterns through its multi-layer architecture, rendering it suitable for both linear and non-linear problems. Detailed parameters for each model are presented in Table 1.

### 2.3. Multioutput Prediction

The MultiOutputClassifier class from the Scikit-learn library was employed in this study to handle multi-label classification, with the goal of predicting resistance to three different drugs simultaneously. This approach allowed for efficient multi-label classification, as it enabled the models to predict all outputs at once without the need for separate training for each drug.

Several ML models were used as base estimators within the MultiOutputClassifier. Specifically, XGBoost (XGBClassifier), LightGBM (LGBMClassifier), Gradient Boosting (GradientBoostingClassifier), and a neural network model (MLPClassifier) were applied. The models were configured with optimized hyperparameters, such as a high number of estimators (2000) and a learning rate of 0.001, to ensure robust performance. The use of MultiOutputClassifier ensured that predictions were made efficiently across all three drugs for each model, while also capturing any potential correlations between drug resistance patterns.

### 2.4. Performance Metrics

The confusion matrix played a crucial role in evaluating the performance of the models, particularly in detecting drug resistance. It distinguishes between true positives, true negatives, false positives, and false negatives, and optimal results are reflected when the majority of values are concentrated along the diagonal of the matrix. To provide a more detailed evaluation, several metrics were calculated based on the confusion matrix.

#### 2.4.1. Accuracy

Accuracy measures the fraction of correct predictions (both true positives and true negatives) made by the model across the entire dataset. This metric is useful when the dataset is balanced between positive and negative cases.

(1) $$\mathrm{Accuracy}=\frac{\mathrm{True}\;\mathrm{Positives}+\mathrm{True}\;\mathrm{Negatives}}{\mathrm{Total}\;\mathrm{Samples}}$$

#### 2.4.2. Sensitivity (Recall or True Positive Rate)

Sensitivity evaluates the model’s ability to correctly identify positive cases, making it particularly important for detecting drug resistance.

(2) $$\mathrm{Sensitivity}=\frac{\mathrm{True}\;\mathrm{Positives}}{\mathrm{True}\;\mathrm{Positives}+\mathrm{False}\;\mathrm{Negatives}}$$

#### 2.4.3. Specificity (True Negative Rate)

This metric measures the model’s ability to correctly identify negative cases, or those that are susceptible to the drugs. High specificity indicates the model’s effectiveness in avoiding false positives.

(3) $$\mathrm{Specificity}=\frac{\mathrm{True}\;\mathrm{Negatives}}{\mathrm{True}\;\mathrm{Negatives}+\mathrm{False}\;\mathrm{Positives}}$$

#### 2.4.4. Precision

Precision represents the proportion of true positive predictions among all positive predictions made by the model. It is a key metric for evaluating the accuracy of the model in identifying drug-resistant isolates.

(4) $$\mathrm{Precision}=\frac{\mathrm{True}\;\mathrm{Positives}}{\mathrm{True}\;\mathrm{Positives}+\mathrm{False}\;\mathrm{Positives}}$$

#### 2.4.5. F1-Score

The F1-score combines both precision and sensitivity into a single metric, providing a balanced view of the model’s performance, especially when the class distribution is imbalanced. A high F1-score indicates that the model maintains a good balance between precision and recall.

(5) $$F1-\mathrm{Score}=\frac{2\times (\mathrm{Precision}\times \mathrm{Sensitivity})}{\mathrm{Precision}+\mathrm{Sensitivity}}$$

These metrics were calculated for each model to provide a comprehensive assessment of their performance in predicting drug resistance. Confusion matrices for each model and dataset are provided in the Supplementary Materials.

### 2.5. Statistical Analysis Method

To assess the statistical significance of differences in model performance, a Kruskal–Wallis test was conducted. This non-parametric test was chosen because it is suitable for comparing performance metrics across multiple models, especially when the data do not follow a normal distribution, as is often the case with machine learning performance metrics. The test was performed on the four models (XGBC, LGBC, GBC, and ANN), using sensitivity, specificity, precision, F1-score, and accuracy as the performance metrics. The Kruskal–Wallis test was selected because it allows for the evaluation of whether there are significant differences in model performance without making assumptions about the underlying distribution of the data, which is crucial when dealing with complex datasets like those used in this study. Based on these results, follow-up analyses were performed to examine the models further, focusing on those with comparable performance (excluding the ANN model in subsequent analyses).

## 3. Results

### 3.1. Data Overview

This study demonstrated the ability to predict drug resistance in *M. tuberculosis* by training a model on a matrix incorporating genome-wide mutations and drug resistance data in binary form (1 for presence/resistance, 0 for absence/susceptibility). Mutations included in the training matrix were found in both intergenic regions and coding regions previously associated with drug resistance, as well as in genes not previously implicated in this context. This expanded dataset allows for a more comprehensive analysis of mutations and their potential impact on drug resistance.

### 3.2. Model Performance and Statistical Analysis

Four machine learning models—XGBC, LGBC, GBC, and ANN—were implemented to predict resistance to E, H, and R. The XGBC model, trained on the original dataset, achieved high sensitivity values of 0.97, 0.90, and 0.94, and specificity values of 0.97, 0.99, and 0.96 for E, H, and R, respectively.

The LGBC model also performed well, achieving sensitivity values of 0.85, 0.90, and 0.94 for E, H, and R, respectively. While slightly lower than XGBC in sensitivity for ethambutol, LGBC achieved competitive specificity values of 0.98, 0.98, and 0.94 for the same drugs. Similarly, the GBC model demonstrated consistent performance with sensitivity values of 0.80, 0.86, and 0.94 for E, H, and R, and it excelled in specificity, achieving 1.00, 0.94, and 0.98 for the same drugs. The ANN model, however, exhibited poorer results, particularly in sensitivity, which was significantly lower than the other models.

**Figure 4:**
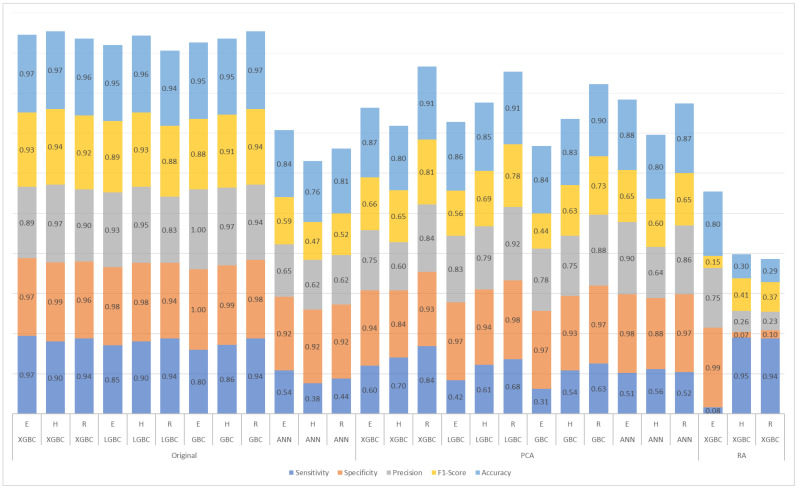
Results for ethambutol (E), isoniazid (H), and rifampicin (R) of the models trained with the original dataset, the dataset reduced by principal component analysis (PCA), and the dataset with arbitrary reduction (RA) with the most important features in the XGBC model.

To address the dataset imbalance favoring drug-susceptible isolates, sensitivity, specificity, and F1-score metrics were analyzed and compared (Figure 4). Models trained on the original dataset consistently outperformed those trained on a dataset reduced by principal component analysis (PCA), indicating better classification accuracy without dimensionality reduction.

A Kruskal–Wallis test was conducted on all four models (XGBC, LGBC, GBC, and ANN) to evaluate significant differences in performance across various metrics (sensitivity, specificity, precision, F1-score, and accuracy) (Table 2). Statistically significant differences were found in specificity (*p* = 0.043), precision (*p* = 0.047), and accuracy (*p* = 0.039), primarily due to the lower performance of the ANN model (Figure 4).

Given the ANN model’s suboptimal performance, a secondary analysis was conducted, excluding it to focus on the remaining models (XGBC, LGBC, and GBC) (Table 2). In this follow-up test, no significant differences were observed across any evaluated metric (*p* > 0.17 across all metrics), suggesting that the ANN model’s lower sensitivity (0.55 compared to values above 0.90 for the other models) was the primary factor driving statistical significance in the initial analysis.

### 3.3. Feature Importance

Extracting the most important features from the XGBC model enabled a direct comparison with resistance mutations cataloged by the WHO for *M. tuberculosis*. Table 3 highlights that a significant portion of the features identified by the XGBC model correspond to mutations listed in the WHO catalog, specifically within resistance-associated genes such as katG, rpoB, rpsL, and gyrA.

The table provides detailed information about each mutation, including its index in the training matrix, genomic position (G. Position), associated gene, and whether it is present in the WHO catalog (indicated by a checkmark in the “Catalog” column). Additionally, the “Feature Importance” column quantifies how often each mutation was utilized across the decision trees within the XGBC model for predicting resistance to E, H, and R.

Further analysis of the XGBC-PCA model, which incorporated principal components derived from the PCA-transformed dataset, revealed a similar alignment with WHO-reported resistance mutations. Table 4 details the contributions of the top five PCs for each drug’s classification. Each PC represents a weighted combination of mutations, and the table lists the top five contributing mutations along with their genomic positions, associated genes, catalog status, and “PC Importance” values.

These observations indicate that the XGBC model emphasizes mutations cataloged by the WHO as critical markers of resistance. However, the XGBC-PCA model, while retaining key features within its top PCs, may obscure individual mutations by combining them into broader components, potentially explaining the model’s reduced sensitivity for H and R.

The disparity in sensitivity and specificity between the models underscores the influence of dataset characteristics and feature selection on model performance. For H and R, the XGBC-RA model demonstrated high sensitivity values (0.95 and 0.94, respectively), likely due to its emphasis on critical resistance mutations. However, this sensitivity came at the cost of specificity (0.07 and 0.10), possibly because the reduced feature set underrepresented mutations relevant to susceptibility. For E, the model exhibited the opposite pattern, achieving high specificity (0.99) but low sensitivity (0.08) (Figure 4).

This trade-off reflects the inherent class imbalance in the dataset, where susceptible samples were more prevalent than resistant ones. In this context, the RA dataset’s reduced feature set prioritized resistance-related mutations, which improved sensitivity for resistant cases but compromised the model’s ability to identify susceptible ones reliably. These findings emphasize the importance of balancing sensitivity and specificity when designing predictive models for clinical applications.

## 4. Discussion

This study demonstrates the potential of ML models, particularly the XGBC model, to predict drug resistance against R, H, and E in *M. tuberculosis* using a binary matrix of genome-wide mutations. The XGBC model performed as well as or better than direct association methods and other ML models reported in previous research [8,9,10,11,12]. However, it is important to acknowledge that other models, such as LGBC and GBC, also showed strong performance, with results comparable to those of XGBC. Notably, there was no significant difference in performance among these three models, further supporting the effectiveness of decision tree-based methods for this type of binary training and classification task.

Despite the strong performance of XGBC with the original dataset, its performance significantly decreased when trained on the RA dataset, which was derived by selecting the most important features identified by XGBC. The disparity in sensitivity and specificity between the models trained on the original and RA datasets underscores the influence of feature selection on model performance. For H and R, the XGBC-RA model demonstrated high sensitivity values (0.95 and 0.94, respectively), likely due to its focus on critical resistance mutations. However, this came at the cost of specificity (0.07 and 0.10), suggesting that the reduced feature set underrepresented mutations relevant to susceptibility. For E, the XGBC-RA model exhibited the opposite pattern, achieving high specificity (0.99) but low sensitivity (0.08), which highlights the trade-off between sensitivity and specificity when reducing features.

These results can be attributed to the inherent class imbalance in the dataset, where susceptible samples are more prevalent than resistant ones. In this scenario, the model becomes biased towards predicting the majority class, leading to a trade-off between sensitivity and specificity depending on the drug. The RA dataset, with its reduced feature set, probably emphasized critical mutations to predict resistance, thereby improving the sensitivity for resistant cases. However, this emphasis may have come at the cost of reducing the model’s ability to accurately identify susceptible cases, particularly for INH and RIF, where relevant mutations for susceptibility may not have been adequately represented in the reduced dataset.

The performance of models trained on datasets with dimensionality reduction (using PCA) also exhibited decreased sensitivity and specificity compared to those trained on the original dataset. This further reinforces the challenges of using PCA with binary data, as while PCA can simplify data, it may not always preserve the critical features necessary for accurate prediction. The reduced dimensionality, both in the RA and PCA datasets, may have led to the loss of mutations important for susceptibility classification, ultimately compromising the model’s ability to accurately predict resistant and susceptible cases.

Significant differences were observed in the statistical analysis performed when comparing the performance metrics among the four models (Table 2). The ANN model exhibited lower sensitivity, likely contributing to these observed differences. However, it is essential to highlight that the ANN needed to be thoroughly optimized in this study; instead, the primary focus was on enhancing the performance of the other models, specifically XGBC, LGBC, and GBC. It has been demonstrated, however, that ANNs can achieve good predictive results in drug resistance studies when thoroughly optimized and explored in depth [16].

As a result, when the ANN was excluded from the analysis, the remaining models (XGBC, LGBC, and GBC) demonstrated no significant differences in their performance metrics. This indicates that, while the ANN may have impacted the overall comparative results, the performance of the other models remained consistent.

Despite the lack of significant differences among them, the sensitivity values of the XGBC model still showcased its predictive solid power for drug resistance, reinforcing its position as a preferred choice for clinical applications.

Despite a reduced dataset (847 IDs selected from 12,289 original datasets), the XGBC model achieved favorable results, suggesting that the selected isolates may contain significant and representative mutations critical for predicting drug resistance. This implies that relevant and informative features can still drive effective model performance even with a smaller dataset. However, increasing computational power and using a more comprehensive training matrix could enhance the accuracy and generalizability of the model. Larger datasets would likely capture a wider variety of genetic diversity and drug resistance profiles, leading to more robust predictions.

The study also highlights the importance of intergenic regions in predicting drug resistance, with the XGBC model incorporating these regions into its classification. This aligns with previous studies that identified significant mutations in intergenic regions, such as embC-embA and oxyR-ahpC, related to resistance to E and H [17,18]. These regions, though non-coding, could harbor mutations that affect the regulation of resistance genes, possibly influencing drug susceptibility. Given the complexity of the *M. tuberculosis* genome, the inclusion of intergenic mutations in future analyses could enhance the accuracy of resistance predictions, providing a more comprehensive understanding of resistance mechanisms. Further investigation into these regions would be valuable in improving predictive models and clinical applications.

As the field evolves, using more advanced ML techniques and algorithms could help make better use of larger datasets, allowing models to identify more detailed patterns related to drug resistance. This would improve their predictive accuracy. While this study highlights the effectiveness of the XGBC model, it also emphasizes the need for larger datasets and improved computational resources to enhance performance. Future research could also benefit from more complex datasets that take into account mutation types, as well as nucleotide changes, which would provide deeper insights into the bacterial mechanisms behind drug resistance. This will be crucial for applying these models in clinical settings. This study not only shows the potential of ML for predicting drug resistance but also provides insights into the genomic factors involved, reinforcing the need for better computational resources and broader datasets in future studies.

## 5. Conclusions

The study shows that a machine learning model called XGBC, which uses a binary training matrix containing information on drug resistance and genome-wide mutation data in *M. tuberculosis*, is highly accurate in predicting drug resistance. It performs better than other methods previously reported. While the XGBC model is a promising tool for classifying drug resistance, there is potential for further refinement and optimization, which presents opportunities for future research. This advancement in the fight against drug-resistant TB offers ML models that can accurately predict resistance patterns, potentially improving treatment strategies and outbreak prevention efforts.

The research establishes a machine learning framework that predicts first-line drug resistance in *M. tuberculosis*, particularly for R, H, and E. The XGBC model demonstrates high specificity and sensitivity, showing its potential as a valuable tool in combating drug-resistant TB. By enhancing our understanding of resistance mechanisms and identifying connections between cases, this work significantly contributes to public health efforts. Implementing XGBC models and considering intergenic mutations and previously unrecognized genes could lead to more efficient strategies in combating drug resistance in TB. Leveraging these insights can enhance our understanding of resistance mechanisms and improve treatment outcomes, ultimately addressing the critical challenge of drug-resistant TB.

## Figures and Tables

**Table 1:** Parameters of the Machine Learning Models Implemented in the Study (XGBC, LGBC, GBC, and ANN).

Classifier	Parameters
**XGBC**	objetive = binary:logistic
	max_depth = 6
	alpha = 15
	learning_rate = 0.001
	n_estimators = 2000
	random_state = 66
	scale_pos_weight = 4
**LGBC**	objetive = binary:logistic
	n_estimators = 2000
	random_state = 66
	learning_rate = 0.001
	scale_pos_weight = 4
**GBC**	n_estimators = 2000
	learning_rate = 0.001
	max_depth = 6
	random_state = 66
**ANN**	hidden_layer_sizes = (100,100,100,3)
	activation = "relu"
	solver = "adam"
	alpha = 0.001
	batch_size = ’auto’
	learning_rate_init = 0.001
	max_iter = 1000
	random_state = 66

**Table 2:** Kruskal–Wallis test for performance metrics across prediction models (XGBC, LGBC, GBC, and ANN; XGBC, LGBC, and GBC).

Metric	XGBC	LGBC	GBC	ANN	*p*-Value (All Models)	*p*-Value (XGBC, LGBC, GBC)
Sensitivity	0.97, 0.90, 0.94	0.85, 0.90, 0.94	0.80, 0.86, 0.94	0.54, 0.38, 0.44	0.0548	0.3247
Specificity	0.97, 0.99, 0.96	0.98, 0.98, 0.94	1.00, 0.99, 0.98	0.92, 0.92, 0.92	0.0431	0.2241
Precision	0.89, 0.97, 0.90	0.93, 0.95, 0.83	1.00, 0.97, 0.94	0.65, 0.62, 0.62	0.0472	0.2356
F1-Score	0.93, 0.94, 0.92	0.89, 0.93, 0.88	0.88, 0.91, 0.94	0.59, 0.47, 0.52	0.0605	0.3839
Accuracy	0.97, 0.97, 0.96	0.95, 0.96, 0.94	0.95, 0.95, 0.97	0.84, 0.78, 0.81	0.0387	0.1710

**Table 3:** Key features used in the XGBC model for ethambutol (E), isoniazid (H), and rifampicin (R).

XGBC														
**E**					**H**					**R**				
**Mutation**	**G. Position**	**Gen**	**Catalog**	**Feature Importance**	**Mutation**	**G. Position**	**Gen**	**Catalog**	**Feature Importance**	**Mutation**	**G. Position**	**Gen**	**Catalog**	**Feature Importance**
14116	761143	rpoB	✓	1729	37159	2155161	katG	✓	2000	14116	761143	rpoB	✓	2000
14105	761095	rpoB	✓	1729	14116	761143	rpoB	✓	1775	14105	761095	rpoB	✓	2000
33509	1955515	Rv1729c		1601	14105	761095	rpoB	✓	1711	14110	761115	rpoB	✓	1737
203	7570	gyrA	✓	1450	29006	1673340	Intergenic		1621	37159	2155161	katG	✓	1510
37159	2155161	katG	✓	1397	49083	2726131	Intergenic		1516	1397	48443	Rv0044c		1206
47752	2637194	Intergenic		1382	14110	761115	rpoB	✓	1299	7817	386402	Rv0318c		1018
14781	799050	Rv0698		1077	8561	423722	hspR		1092	1054	36346	bioF2		922
55982	3096203	Rv2787		1004	25877	1472189	rrs	✓	1031	50450	2801781	PE_PFRS43		849
26321	1501584	Intergenic		929	68243	3878152	rpoA		1026	49083	2726131	intergenic		825
36805	2154002	katG		883	29993	1728470	Intergenic		838	6289	292515	fadA2		721
50450	2801791	PE_PGRS43		614	5708	262407	Rv0219		717	14517	781662	rpsL	✓	490
74508	4247249	embB	✓	603	70630	4037025	PE_PGRS59		541	14781	799050	Rv0698		424
1054	36346	bioF2		589	69123	3940775	PE_PGRS55		445	50436	2801205	Intergenic		398
14110	761115	rpoB	✓	550	14247	766466	rpoC		423	26411	1506974	rphA		324
3160	132551	PE_PGRS1		504	59556	3347434	Rv2990c		409	9557	485223	fadD30		309
60495	3414761	nrdH		496	21753	1224048	phoH2		345	43282	2357046	PE_PFRS36		294
15351	835654	Rv0745		459	53893	3021823	Intergenic		306	1609	57537	Rv0052		281
7722	381610	Rv0312		424	17431	968250	cspB		217	2382	93274	hycD		275
66257	3741177	PE_PGRS50		347	78223	4377951	eccC2		195	77648	4359079	espK		263
17733	988380	Rv0888		347	6358	295561	fadE5		195	54150	3037139	fadE20		263

The “Mutation” column represents the index position of each mutation in the training matrix, while “G.Position” specifies the genomic location of the mutation within the *M. tuberculosis* genome. The associated gene is identified by its name, or labeled as “Intergenic” if the mutation is located in a non-coding region. The “Catalog” column indicates whether the mutation is included in the WHO catalog of drug resistance-associated mutations [15], providing a reference for its relevance in predicting resistance. “Feature Importance” reflects the contribution of each mutation in the model’s prediction, highlighting its utilization in decision trees.

**Table 4:** Key features used in the XGBC-PCA model for ethambutol (E), isoniazid (H), and rifampicin (R).

XGBC														
**E**					**H**					**R**				
**PC**	**G.Position**	**Gen**	**Catalog**	**PC Importance**	**PC**	**G.Position**	**Gen**	**Catalog**	**PC Importance**	**PC**	**G.Position**	**Gen**	**Catalog**	**PC Importance**
11	4046007	Rv3603c		1715	46	2155168	katG	✓	1878	46	2155168	katG	✓	2242
	484005	fadD30				761155	rpoB	✓			761155	rpoB	✓	
	1529133	Rv1358				454295	Rv0376c				454295	Rv0376c		
	1446733	argS				3173107	Intergenic				3173107	Intergenic		
	1151304	kdpD				3487860	Rv2876				3187860	Rv2876		
0	2177366	fadD31		959	39	1753519	Intergenic		1028	11	4046007	Rv3603c		1199
	3962187	Rv3525c				3308310	Rv2955c				484005	fadD30		
	1849051	lysX				3186860	dipZ				1529133	Rv1358		
	2354791	pafB				2634282	Intergenic				1446733	argS		
	1285001	pimE				1443428	Intergenic				1151304	kdpD		
14	2976564	Intergenic		906	11	4046007	Rv3603c		907	44	2155168	katG	✓	885
	3969423	PPE61				484005	fadD30				2220512	Rv1977		
	2881382	Intergenic				1529133	Rv1358				3186860	dipZ		
	3901234	Intergenic				1446733	argS				761155	rpoB	✓	
	1914779	tyrS				1151304	kdpD				4008747	hsaB		
3	2288085	Rv2042c		789	81	2155168	katG	✓	882	89	2155168	katG	✓	853
	2288844	pncA				340372	PPE3				2528971	Rv2254c		
	2288883	pncA				781687	rpsL	✓			1313337	intergenic		
	2288867	pncA				2220512	Rv1977				3878547	intergenic		
	2288856	pncA				3186860	dipZ				1313338	intergenic		
30	2155168	katG	✓	757	15	1164571	Intergenic		858	30	2155168	katG	✓	790
	761155	rpoB	✓			2168742	PPE35				761155	rpoB	✓	
	3189481	Intergenic				2700	dnaN				3189481	intergenic		
	4318188	Intergenic				8147	gyrA	✓			4318188	intergenic		
	628174	galE3				1444134	Rv1290c				628174	galE3		

The columns represent the principal components (PCs) derived from the PCA-transformed dataset, each associated with a combination of mutations. “G.Position” indicates the genomic position of the mutations within the *M. tuberculosis* genome, while the “Gene” column identifies the gene where the mutation occurs or is labeled as “Intergenic” if located in a non-coding region. The “Catalog” column shows whether the mutation is listed in the WHO catalog of drug resistance-associated mutations [15], providing context for its relevance in predicting resistance. “PC Importance” reflects the contribution of each principal component to the model’s prediction.

## Data Availability

The data supporting the findings of this study are available upon reasonable request from the corresponding author. Due to privacy and ethical considerations, access to certain portions of the dataset may be restricted.

## Supplementary Materials

The following supporting information can be downloaded at: https://www.mdpi.com/article/10.3390/diagnostics15030279/s1.
